# Uterine torsion complicated by severe placental abruption in the second trimester: a case report and literature review

**DOI:** 10.1186/s12884-023-05377-z

**Published:** 2023-01-21

**Authors:** Shuhua Liu, Linlin Zhou, Bing Song, Dehong Liu, Chenmin Zheng, Xiumei Wu, Zhaolian Wei, Xianxia Chen

**Affiliations:** 1 Department of Obstetrics and Gynecology, Anhui Province Maternity and Child Health Hospital, Hefei, China; 2 Department of Obstetrics and Gynecology, the First Affiliated Hospital of Anhui Medical University, Hefei, China; 3 First Clinical College, Anhui Medical University, Hefei, China

**Keywords:** Uterine torsion, Placental abruption, Rare cause

## Abstract

**Background:**

Uterine torsion is a rare obstetric event that can occur during pregnancy and is difficult to diagnose. Its occurrence may lead to serious adverse pregnancy outcomes.

**Case introduction:**

The patient was a 33-year-old woman at 30^+ 5^ weeks’ gestation with a singleton pregnancy. The pregnancy course, including fetal growth, and prenatal examinations were regular. Except for a small amount of vaginal bleeding in early pregnancy and treatment with progesterone, there were no prenatal abnormalities, and the patient denied any trauma or sexual history. The patient was admitted to the emergency department with persistent severe pain in the lower abdomen and slight vaginal bleeding during night sleep. Abdominal pain started two hours prior to admission and was accompanied by nausea, vomiting, and dizziness. Examination revealed positive abdominal tenderness, high uterine tone, and no significant intermittent period of uterine contractions, and measurement of the fetal heart rate by means of the nonstress test revealed a rate of 60 beats per minute. Therefore, placental abruption was highly suspected. Subsequently, an emergency cesarean section was performed under general anesthesia. The newborn boy, with Apgar scores of 0–3-4 after birth and weighing 1880 g, was transferred to the neonatal intensive care unit (NICU) and died two days later due to ineffective rescue. After the uterine incision was sutured, the examination revealed that the uterine incision was located on the posterior wall of the uterus, and the uterus was twisted 180° to the right. The diagnosis after cesarean section was 180° uterine torsion to the right, severe placental abruption, and severe neonatal asphyxia. On the fifth day after surgery, the patient recovered and was discharged from the hospital.

**Conclusions:**

Posterior uterine incision cesarean section may be performed in unexpected circumstances and is also feasible as a safe option for resetting if torsion is not complete. Abdominal pain during pregnancy is less likely to be diagnosed as uterine torsion, which often leads to premature birth, fetal asphyxia, placental abruption, and even perinatal death. Therefore, for abdominal pain during pregnancy, obstetricians should consider the possibility of uterine torsion.

## Background

Physiological uterine torsion occurs when the sigmoid ring is located on the left side of the peritoneal cavity during pregnancy and does not exceed 45° [1]. Uterine torsion is defined as a rotation of more than 45° about the long axis of the uterus, usually to the right [2, 3]. The pivot point is mostly at the level of the isthmus [1].

First reported in 1861 [4], uterine torsion during pregnancy is a rare, unexpected obstetric emergency [5, 6], and may be a “never seen in a lifetime” diagnosis for most obstetricians. It is almost always diagnosed during cesarean section [7, 8], and it can occur at any stage of pregnancy. Although uterine torsion is extremely rare, it can cause serious maternal and infant complications [9] and occur again in subsequent pregnancies [10].

This report describes a case involving severe placental abruption and neonatal asphyxia caused by uterine torsion with the death of the newborn two days later due to ineffective rescue.

## Case presentation

The patient was a pregnant woman (gravida 1, para 0) aged 33 years with a singleton pregnancy at 30^+ 5^ weeks of gestation. The pregnancy course, including fetal growth, and prenatal examination were regular. Except for a small amount of vaginal bleeding in early pregnancy and treatment with progesterone treatment, there were no prenatal abnormalities, and the patient denied any trauma or sexual history.

**Fig. 1:**
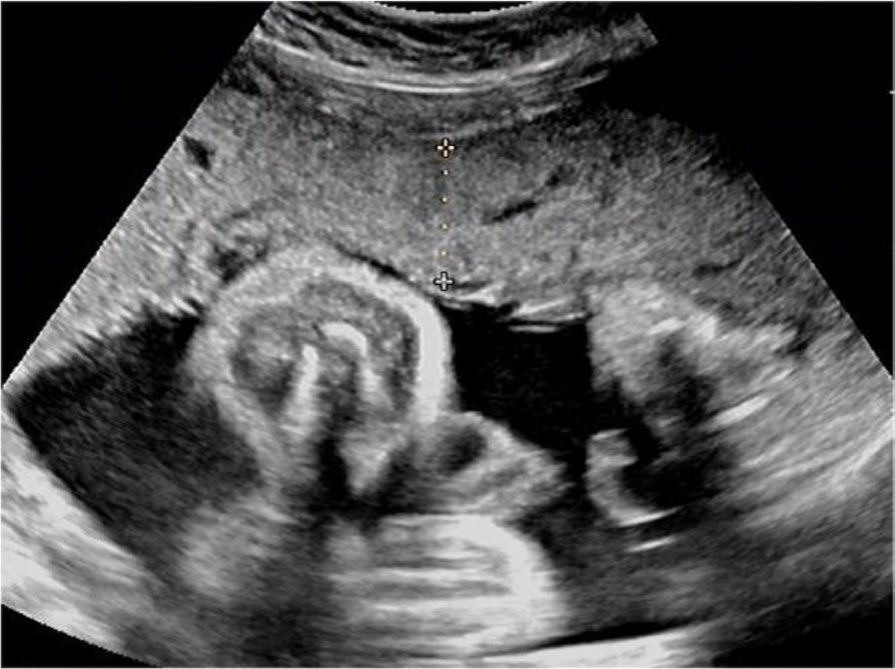
On the afternoon of the day before this visit, The placenta was attached to the uterine anterior wall, with a placental thickness of 23 mm

On the afternoon of the day before this visit, obstetric examination and ultrasonography were performed in our hospital to evaluate the condition of the fetus and placenta. Ultrasonography showed a single fetus and umbilical cord around the neck, a normal amniotic fluid volume, and fetal biological measurements that were appropriate for 32 weeks of gestation. The placenta was attached to the uterine anterior wall, with a placental thickness of 23 mm (Fig. 1).

The patient was admitted to the emergency department due to persistent severe pain in the lower abdomen. Abdominal pain began two hours before admission and was accompanied by nausea, vomiting, and dizziness. The physical examination showed a T of 36.5 °C, P of 100 beats per minute, R of 20 beats per minute, BP of 112/70 mmHg, clear consciousness, and a pale complexion. Obstetric examination showed that uterine growth was suitable for gestational age, abdominal tenderness was positive, uterine tension was high, uterine contractions were not significantly intermittent, and the fetal heart rate was 60 beats per minute, as detected in a nonstress test. Therefore, placental abruption was highly suspected. An emergency cesarean section was subsequently performed under general anesthesia.

**Fig. 2:**
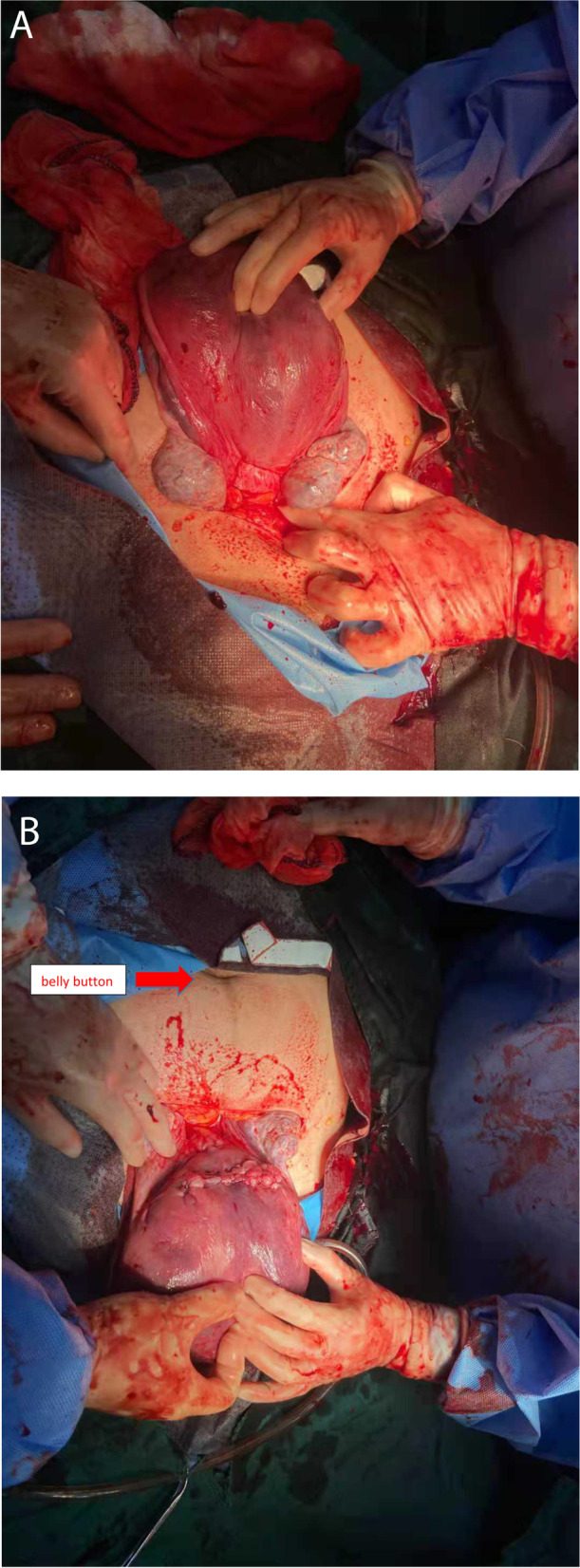
**A**. The anterior part of the uterus after reverse torsion was shown from medial to lateral, with the proper ligament of the ovary, fallopian tube, and uterine round ligament. **B**. The posterior part of the uterus after reverse torsion shows that the uterine incision is a transverse incision of the lower uterine segment, and the red arrow indicates the patient’s belly button

We opened the abdomen horizontally, and the lower part of the uterus was cut horizontally. Then, a baby boy, with Apgar scores of 0–3-4 and weighing 1880 g, was removed and transferred to the NICU; the boy died two days later due to ineffective rescue. The placenta also emerged after the fetus was delivered, and 500 ml of blood clots were found in the uterine cavity. During the operation, the uterus was paralyzed, 20 units of oxytocin (H32025280, China) and 250 μg of Carboprost Tromethamine Injection (H20170146, Pharmacia & Upjohn) were combined into the myometrium to stimulate uterine contractions, and ligation of the ascending branch of the uterine artery was performed to prevent bleeding. After closing the uterine incision, we found that the uterus was twisted 180° to the right and that the incision was located in the posterior wall of the uterus. The anterior part of the uterus after reverse torsion was shown from medial to lateral, with the proper ligament of the ovary, fallopian tube, and uterine round ligament (Fig. 2 A). The uterine incision was located on the posterior wall of the uterus (Fig. 2 B). After surgery, the placenta was sent for pathological examination, which indicated placental abruption. The main postoperative diagnosis was uterine twist to the right by 180°, placental abruption, and severe neonatal asphyxia.

**Fig. 3:**
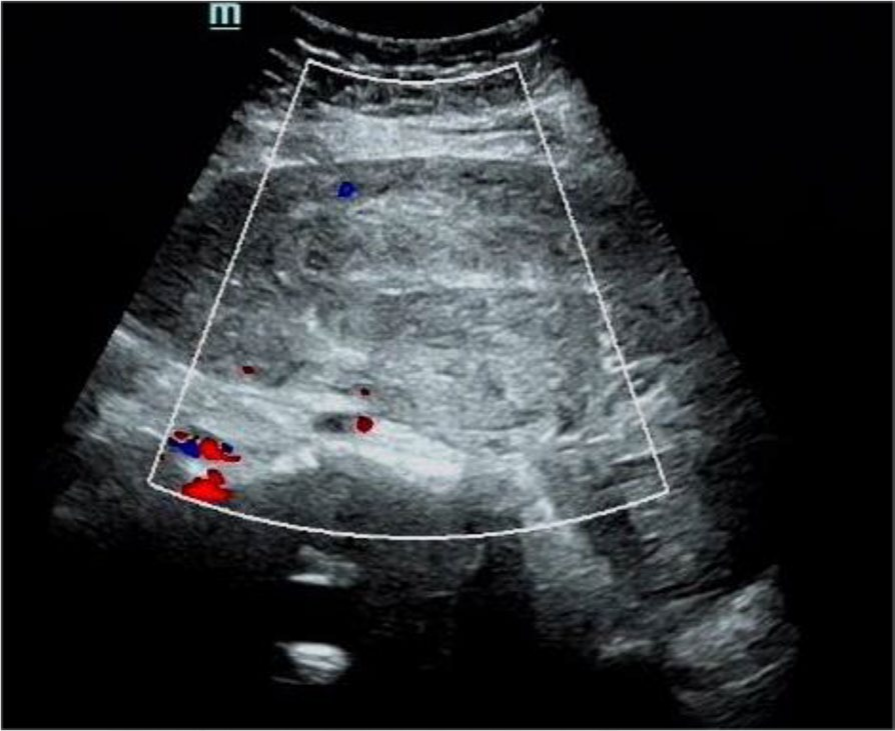
Postoperative ultrasonography showed that the incision of the posterior wall of the uterus had recovered well, the myometrium was continuous, the intrauterine line was clear, and there was no hemorrhage or placental membrane residue in the uterine cavity

The patient was stable after surgery. A hemoglobin concentration of 96 g/liter was measured at emergency admission, and blood gas analysis was performed, with a hemoglobin of 88 g/liter during surgery and 73 g/liter after surgery. Postoperative ultrasonography showed that the incision of the posterior wall of the uterus had recovered well, the myometrium was continuous, the intrauterine line was clear, and there was no hemorrhage or placental membrane residue in the uterine cavity (Fig. 3). The patient was discharged successfully.

## Discussion and conclusions

Uterine torsion was first reported in 1861, but just over 200 cases have been reported in the past 100 years [11]. The etiology of uterine torsion during pregnancy remains unclear. According to reports, the causes of most cases of uterine torsion include congenital or acquired uterine malformations, pelvic tumors or uterine fibroids or adhesions, traction, or abnormal fetal position that may lead to significant uterine asymmetry [7, 12]. For these reasons, we need to screen patients for the above causes in the first trimester and before pregnancy, inform them of the relevant risks, and strengthen education and management during pregnancy to reduce the occurrence of adverse pregnancy outcomes. In addition, pregnancy can exacerbate the congenital and physiological rotation and tilt of the uterus, but most cases still cannot be explained by the above reasons.

In general, symptoms are related to the degree and duration of uterine torsion. Previous reports have shown peritonitis-like symptoms in 16% of patients, urinary tract symptoms in 8%, and asymptomatic symptoms in 11% [13]. Therefore, prenatal diagnosis of uterine torsion is not easy. Uterine torsion is more difficult to suspect in asymptomatic cases [7]. Moreover, most cases are determined only during surgery and sometimes even after the uterine incision has been sutured [14]. The study pointed out that since the normal vagina has an H-shaped structure on magnetic resonance imaging (MRI), an X-shaped structure of the vagina on MRI is a sign of uterine torsion [15]. However, MRI is not an appropriate test in the emergency rescue of mothers and babies. Excessive inspections and time spent on imaging may lead to missed opportunities for rescue efforts.

Although symptoms such as abdominal pain and vaginal bleeding are not specific enough, this case alerts the obstetrician to the possibility of uterine torsion. In this case, we considered the diagnosis of placental abruption and fetal distress based on symptoms, signs and fetal heart rate monitoring. Emergency cesarean section is performed in critical situations without ultrasound evaluation. If the situation is less urgent, we may perform an ultrasound evaluation and compare the previous results, and perhaps a diagnosis of uterine torsion can be confirmed before delivery.

By narrowing the venous lumen, twisting of any uterus-like organ results in a decrease in perfusion. When uterine torsion occurs, the torsional venous lumen narrows, venous blood flow decreases first, and then the blood supply to the uterus decreases, followed by increased pressure in the placental cotyledons leading to fetal distress and placental abruption. Studies have identified factors associated with placental abruption, including maternal asthma, previous cesarean section, cocaine use, endometriosis, chronic hypertension, older maternal age, maternal smoking, use of antiretroviral therapy, low preconception weight, preeclampsia, uterine leiomyoma and cannabis use [16]. Based on this report, uterine torsion, a rare disease, should also be added. Management of perinatal uterine torsion depends on when torsion occurs during pregnancy. In all cases, laparotomy is necessary. When uterine torsion occurs before the fetus is viable, treatment is laparotomy and uterine detorsion.

When the uterus is twisted, the fallopian tubes and ovaries are located anteriorly, there is no normal bladder reflex peritoneum, the lower uterine segment is constricted, the vessels on the surface of the lower segment are extremely hyperemic, and the ureter is closer to the uterus. Agar et al. recommend the use of longitudinal uterine incision in suspected cases of uterine torsion to prevent damage to blood vessels or ureters [17]. However, Albayrak and colleagues have noted that in unexpected situations, when detorsion is not performed, a posterior low percutaneous incision is also a safe option [18]. Similarly, this procedure has also been shown to be practical in this case [18].

Through this case, we learned that uterine torsion causes serious harm to both mothers and babies. Therefore, we should optimize antenatal care and examination and reduce or advise avoidance of sexual activity and heavy physical labor in the second and third trimesters of pregnancy as much as possible [19]. In patients with a history of uterine leiomyomas, preconception resection or intensive monitoring during pregnancy should be considered based on the size and location of the fibroids [19]. In addition, pregnancy care and timely treatment and monitoring of symptoms such as abdominal pain are needed.

## Conclusion

Uterine torsion is an extremely rare obstetric emergency that can easily lead to severe placental abruption, fetal distress and even intrauterine death. Unfortunately, uterine torsion is difficult to diagnose prenatally. Through this report, in the case of unanticipated uterine torsion before surgery, cesarean section incision in the posterior wall of the uterus is also a safe and feasible surgical option. The emergency treatment of placental abruption and newborns should be the focus rather than the cause of uterine torsion.

## Data Availability

The datasets generated and/or analyzed during the current study are not publicly available because they contain information that could identify the research participant, but they are available from the corresponding author on reasonable request.

## Abbreviations

NICU: Neonatal Intensive Care Unit
T: temperature
P: pulse
R: respiration
BP: blood pressure
MRI: magnetic resonance imaging
g: gram
mm: millimeter
